# A fixation-compatible protocol for intracellular and surface marker-based detection of circulating tumor cells in hepatocellular carcinoma

**DOI:** 10.1038/s41598-025-23698-1

**Published:** 2025-11-07

**Authors:** Boqun Zhu, Kanako Yoshida, Harry Luu, Hao Zhang, Joseph V. Passero, Kathy Liu, Selena Y. Lin, Ying-Hsiu Su, Hyunsoo Song, James P. Hamilton, Qingfeng Zhu, Robert A. Anders, Amy K. Kim

**Affiliations:** 1 Department of Gastroenterology and Hepatology, Johns Hopkins University School of Medicine, Baltimore, MD USA; 2 Department of Hepatology, Graduate School of Medicine, Osaka Metropolitan University, Osaka, Japan; 3 Department of Molecular Microbiology and Immunology, Johns Hopkins Bloomberg School of Public Health, Baltimore, MD USA; 4 JBS Science, Inc, Doylestown, PA USA; 5 Baruch S. Blumberg Institute, Doylestown, PA USA; 6 Department of Oncology, Sidney Kimmel Comprehensive Cancer Center, Johns Hopkins University School of Medicine, Baltimore, MD USA; 7 Department of Pathology, Johns Hopkins University School of Medicine, Baltimore, MD USA

**Keywords:** Hepatocellular carcinoma, Biomarkers, Hepatocellular carcinoma

## Abstract

Circulating tumor cell (CTC) detection in hepatocellular carcinoma (HCC) is limited not only by the rarity of CTCs but also by a heavy reliance on cell surface markers such as EpCAM, which are variably expressed or lost during tumor progression. Detecting intracellular markers, such as cytokeratin offers an important complementary and comprehensive strategy but remains technically limited in flow cytometry due to the need for fixation and permeabilization, which often lead to cell loss and surface epitope damage. In this study, we systematically evaluated the feasibility of using fixed samples for flow cytometry, using HepG2 cells, PBMCs, and CTCs from patients with HCC. Our results demonstrate that fixation enabled intracellular staining without compromising cell surface marker detection, even after short-term storage at 4 °C and long-term storage at -80 °C. Fixed samples, particularly fixed unfrozen, exhibited comparable staining performance to fresh samples with only a 7–10% reduction in cell recovery. Clinical validation in HCC patients confirmed successful CTC detection, and tumor-specific CTNNB1 mutations were identified in CTC-derived DNA but not in matched plasma cfDNA. These findings support fixed CTC sample workflows as a reliable and practical approach for CTC analysis in HCC.

**Supplementary Information:**

The online version contains supplementary material available at 10.1038/s41598-025-23698-1.

## Introduction

Hepatocellular carcinoma (HCC) is one of the most common and lethal cancers globally, ranking as the sixth most prevalent cancer and the third leading cause of cancer-related death^1^. Despite its clinical significance, the currently approved surveillance biomarker, alpha-fetoprotein (AFP), has limited sensitivity (40–60%) and provides only indirect evidence of HCC^2^. This highlights an urgent need for more reliable, sensitive, and dynamic biomarkers for early detection and disease monitoring in HCC. Liquid biopsy which is defined as circulating tumor cells (CTCs) or tumor-derived molecules including DNA and RNA collected from blood, urine, or other body fluids, has emerged as a promising alternative, offering a less invasive, more accurate, and dynamic approach to monitoring tumor progression in cancer^3–7^. Successful detection of CTCs not only enhances the understanding molecular characteristics and progression evaluation, but also aids in treatment response evaluation and clinical decision-making, and ultimately, personalized therapy^4,8–11^.

However, the clinical implementation of CTCs remains limited, primarily due to their rarity and the challenges associated with consistent and reproducible biospecimen collection and processing, limiting its reliable detection. To overcome their low abundance, CTC detection workflows require an enrichment step. Current methods for enriching CTCs are commonly classified into two broads, and sometimes overlapping, categories: those that exploit physical properties such as size, density, or deformability, and those that rely on biological characteristics, including the expression of specific surface markers^8,12^. Each method has limitations, such as low purity, low yield, underestimation, or the need for specialized equipment. To address these challenges, combining enrichment strategies that integrate different modalities have been proposed. Detection of CTCs by flow cytometry following either positive or negative enrichment is emerging as a feasible and reliable method^13–19^, enabling the use of multiple targets to enhance sensitivity beyond the more commonly used EpCAM-based approaches. This is particularly important in the context of epithelial-to-mesenchymal transition (EMT), during which CTCs may downregulate or lose EpCAM expression^20^. In HCC, this limitation is pronounced, as only about 35% of CTCs express EpCAM^21^.

Cytokeratin is another reliable and stable epithelial biomarker commonly used for CTC detection across solid tumors, including HCC. However, because it is primarily located in the cytoplasm, its detection by flow cytometry is challenging. Optimal detection of intracellular expression often requires fixation and permeabilization. This step is often avoided due to concerns for cell death and potential degradation of surface epitopes^22^, making routine cytokeratin staining technically challenging. As a result, without optimized protocols, cytokeratin-based detection remains underutilized in flow cytometry workflows, despite its potential to improve CTC identification – particularly in HCC, where surface marker expression such as EpCAM may be variable or lost.

Despite the potential of flow cytometry following initial enrichment for CTC detection, its broader application remains limited by the lack of standardized sample preparation, especially for flow cytometric analysis. Most workflows rely on fresh, viable cells and require immediate downstream analysis, which is time-consuming and logistically challenging – especially for studies with large clinical sample collections. Therefore, improved standardization and operational efficiency are essential for broader implementation.

To overcome these challenges, we developed a streamlined and robust CTC detection protocol optimized for HCC. The method incorporates fixation and permeabilization for flow cytometry, enabling simultaneous staining of intracellular and cell surface markers while minimizing cell loss and preserving both staining quality and CTC detection rates. This workflow is specifically tailored to the characteristics of CTCs in HCC and has demonstrated feasibility in the analysis of clinical samples from HCC patients. As such, it provides an efficient and reproducible workflow for CTC analysis, suitable for both clinical and research applications in liver cancer.

## Results

### Fixation enables intracellular staining with surface marker integrity

To investigate the effect of fixation and permeabilization process on expression of commonly used cell surface and intracellular markers in HCC CTCs study, we compared fixed/permeabilized versus unfixed cells using flow cytometry. We selected intracellular marker Pan-Cytokeratin (PanCK), and cell-surface markers, EpCAM and CD45 and tested them on both HepG2 cells and peripheral blood mononuclear cells (PBMCs).

**Fig. 1:**
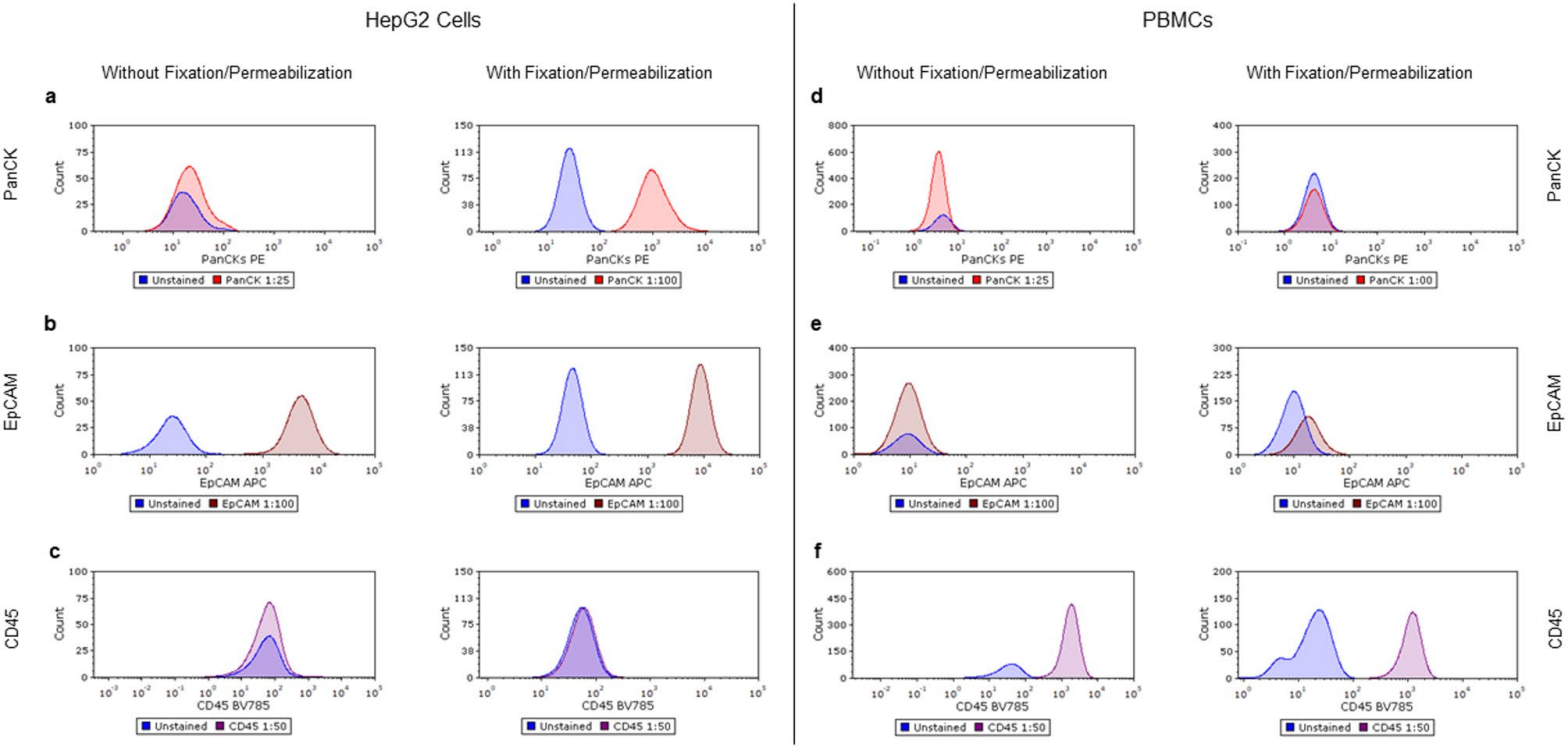
Flow cytometry histograms of PanCK, EpCAM, and CD45 expression in HepG2 cells (left panel) and PBMCs (right panel) with and without fixation/permeabilization. Stained populations are shown as PanCK (red; panel a, d), EpCAM (dark red; panel b, e), and CD45 (purple; panel c, f), with unstained controls shown in blue (panel a – f). (**a**) PanCK expression in HepG2 cells with (right) and without (left) fixation/permeabilization. (**b**) EpCAM expression in HepG2 cells with (right) and without (left) fixation/permeabilization (left) at the same EpCAM concentration. (**c**) CD45 expression in HepG2 cells with (right) and without (left) fixation/permeabilization at the same CD45 concentration. (**d**) PanCK expression in PBMCs with (right) and without (left) fixation/permeabilization. (**e**) EpCAM expression in PBMCs with (right) and without (left) fixation/permeabilization at the same EpCAM concentration. (**f**) CD45 expression in PBMCs with (right) and without (left) fixation/permeabilization (left) at the same CD45 concentration.

For HepG2 cells, fixation/permeabilization enabled clear and robust detection of PanCK at the antibody concentration of 1:100 (Fig. 1a). However, no PanCK signal was detected in the unfixed HepG2 cells, even at the concentration as high as 1:25. Notably, the expression of cell-surface markers EpCAM and CD45, staining remained consistent between fixed/permeabilized and unfixedHepG2 cells (Fig. 1b, c). Similarly, in PBMCs, no PanCK and EpCAM expression was detected under either condition (Fig. 1d, e). CD45 staining was preserved similarly between fixed/permeabilized and unfixed PBMCs (Fig. 1f).

These findings suggest that fixation and permeabilization are essential for detecting intracellular proteins such as PanCK, but do not compromise with the detection of key surface markers such as EpCAM and CD45.

### Simultaneous surface and intracellular staining comparable to serial staining method

As fixation and permeabilization are necessary for intracellular staining, the traditional serial staining protocol requires three steps: cell surface staining, fixation and permeabilization, and then intracellular staining. However, this serial staining approach often results in substantial cell loss caused by repeated washes and centrifugation steps. To minimize cell loss while maintaining staining quality, we compared the serial staining method (3-step method) with a simultaneous staining method (2-step method) using harvested HepG2 cells. In this latter method, cells are first fixed, followed by permeabilization and simultaneous staining of both cell surface and intercellular markers (Supplemental Table 1).

**Fig. 2:**
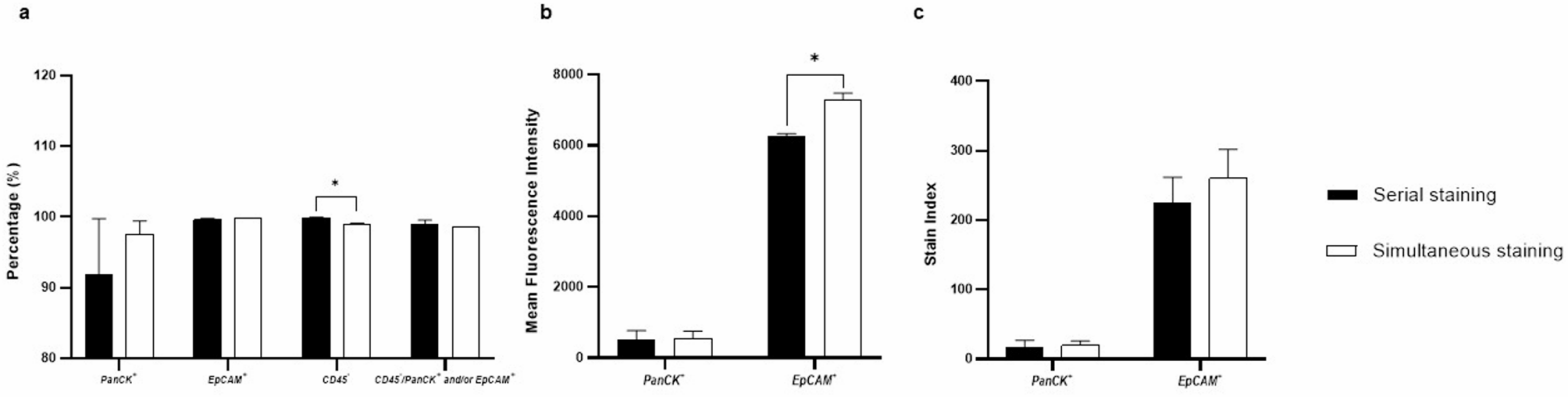
Flow cytometry analysis of PanCK, EpCAM, CD45 staining in HepG2 cells using serial staining and simultaneous staining. (**a**) Comparison of PanCK^+^, EpCAM^+^, CD45^−^ rate, and the “CTC” detection rate (CD45⁻/PanCK⁺ and/or EpCAM⁺). (**b**) Comparison of mean fluorescence intensity (MFI) of PanCK and EpCAM expression after staining. (**c**) Comparison of stain index of PanCK and EpCAM. This experiment was repeated in three independent replicates. Statistical significance was determined using Wilcoxon rank sum tests [*, *P* < 0.05; **, *P* < 0.01; ***, *P* < 0.001; ****, *P* < 0.0001].

The simultaneous staining method demonstrated a slightly lower CD45 negativity rate (98.96%, 98.79% − 99.12%) compared to the serial staining method (99.86%, 99.75% − 99.96%; *P* = 0.0286, *n* = 3), with no significant difference in PanCK positivity rate and EpCAM positivity rate (Fig. 2a). Although the differences in CD45 staining were statically significant, the differences between the two groups are not clinically meaningful when applied for CTC detection.

In addition to individual antibody staining, one sample from each method was stained with all three antibodies to simulate a real CTCs sorting scenario. We compared the detection rate of “CTC”, defined as CD45⁻ cells that were positive for either PanCK, EpCAM, or both (CD45⁻/PanCK⁺ and/or EpCAM⁺ cells). No difference in the “CTC” detection rate was observed between the two methods when using the combined antibody staining (Fig. 2a).

Further analysis showed that the simultaneous staining method resulted in a higher mean fluorescence intensity (MFI) of EpCAM (7234.00, 7097.50–7545.07) compared to the serial staining method (6264.00, 6154.51–6431.01; *P* = 0.0286, *n* = 3), while no significant difference was observed for PanCK MFI or the stain index (SI) of either PanCK and EpCAM (Fig. 2b and c). The lower EpCAM MFI observed with the serial staining method may be due to repeated washes and centrifugation and subsequent fixation/permeabilization.

Therefore, these findings indicate that the simultaneous staining method is comparable to the traditional serial staining method, with the advantage of improved EpCAM staining performance and reduced cell loss – making it a more practical option for CTC analysis in HCC.

### Fixed unfrozen sample Preparation shows comparable cell detection and staining performance to fresh sample

Freshly collected peripheral blood samples are generally preferred for CTC detection, though cryopreserved blood samples have also been used^23^. Meanwhile, fixed cells, which preserve cell morphology and characteristics more stably than fresh or cryopreserved cells, can be an alternative option for long-term storage and scheduled downstream analysis. Therefore, we compared the staining effects of cell surface and intracellular markers across four different sample preparation methods - fresh sample, cryopreserved sample, fixed frozen sample, and fixed unfrozen sample - using HepG2 cells (Supplemental Fig. 1 and Supplemental Table 2) and PBMC samples from healthy donors (Supplemental Table 3).

**Fig. 3:**
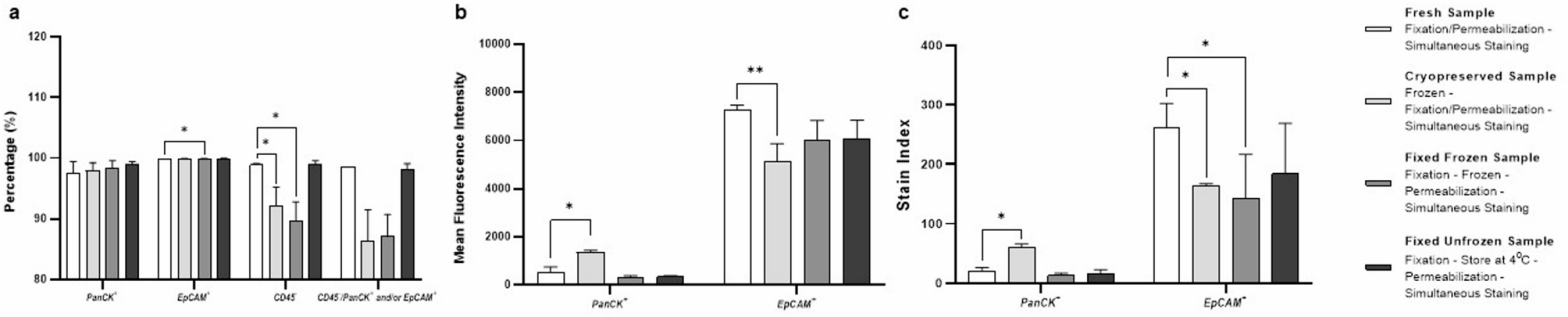
Flow cytometry analysis of PanCK, EpCAM, CD45 staining in HepG2 cells with four sample preparation methods: fresh, cryopreserved, fixed frozen and fixed unfrozen. (**a**) Comparison of PanCK^+^, EpCAM^+^, CD45^−^ rate, and the “CTC” detection rate (CD45⁻/PanCK⁺ and/or EpCAM⁺) across four sample preparation methods. (**b**) Comparison of mean fluorescence intensity of PanCK and EpCAM across four sample preparation methods. (**c**) Comparison of stain index of PanCK and EpCAM across four sample preparation methods. This experiment was repeated in three independent replicates. Statistical significance was determined using Dunn’s multiple comparisons tests after Kruskal-Wallis tests [*, *P* < 0.05; **, *P* < 0.01; ***, *P* < 0.001; ****, *P* < 0.0001].

Regarding individual antibody staining, there was no difference in the PanCK positivity rate across the four groups of HepG2 cells (Fig. 3a). For EpCAM staining, only the fixed frozen HepG2 cells (99.91%, 99.89% − 99.94%) exhibited a significantly higher positivity rate compared to the fresh HepG2 cells (99.83%, 99.80% − 99.87%; *P* = 0.0286) (Fig. 3a). In terms of CD45 staining on HepG2 cells, both the cryopreserved and the fixed frozen HepG2 cells showed slightly higher proportion of CD45 positive staining compared to the fresh HepG2 cells, as reflected in significantly lower CD45 negativity rates (*P* = 0.0286 and 0.0347, respectively). This indicates a higher likelihood of false positive CD45 staining in these groups. In contrast, the fixed unfrozen HepG2 cells showed no significant difference compared to the fresh HepG2 cells (Fig. 3a).

As for the “CTC” detection rate across the four groups, no significant difference was observed between the fresh HepG2 cells and the other three groups (*P* = 0.1238). The cryopreserved and fixed frozen HepG2 cells exhibited a lower detection rate than fresh cells (Median: 86.34%, 87.10% vs. 98.58%, respectively) but not statistically significant (*P* = 0.333 and 0.333, respectively). (Fig. 3a).

In terms of IF staining quality, cryopreserved HepG2 cells showed a significantly higher mean fluorescence intensity (1339.00, 1302.98–1485.25; *P* = 0.0286, *n* = 3) and stain index (SI: 58.65, 55.09–68.40; *P* = 0.0286, *n* = 3) of PanCK compared to fresh HepG2 cells (MFI: 510.60, 358.46–785.74; SI: 18.98, 15.18–27.50) (Fig. 3b, c), while the fixed frozen and fixed unfrozen HepG2 cells showed no difference from the fresh HepG2 cells. Similar to PanCK staining, only cryopreserved HepG2 cells showed a significant difference in EpCAM staining from the fresh HepG2 cells, both the mean fluorescence intensity (MFI: 5136.00, 4435.18–5803.73; *P* = 0.0089, *n* = 3) and stain index (SI: 165.30, 157.54–165.71; *P* = 0.0286, *n* = 3) of EpCAM were lower than those of the fresh HepG2 cells (MFI: 7234.00, 7097.50–7545.07; SI: 262.90, 219.63–296.03) (Fig. 3b, c). Additionally, the fixed frozen HepG2 cells demonstrated a significantly lower stain index (SI: 140.10, 78.27–211.33, *P* = 0.0286, *n* = 3) compared to the fresh HepG2 cells while the mean fluorescence intensity showed no difference between these two groups (Fig. 3b, c). Only the fixed unfrozen HepG2 cells showed no significant difference compared to the fresh HepG2 cells in either the mean fluorescence intensity or stain index of EpCAM.

For CD45 staining on PBMC samples, the CD45 positivity rate, mean fluorescence intensity (MFI), and staining index (SI) in cryopreserved, fixed frozen, and fixed unfrozen PBMCs were not significantly different from those in fresh PBMCs (Supplemental Table 3). Furthermore, no PBMCs were identified as PanCK or EpCAM positive in any of the four groups. These results suggest that these three sample processing methods do not significantly affect CD45 staining or induce non-specific PanCK/EpCAM binding in PBMCs.

Overall, the cryopreserved HepG2 cells exhibited the most distinct staining performance for PanCK, EpCAM, and CD45 compared to the fresh HepG2 cells. Besides, a significant drop in cell scatter was noticed when cells were fixed after freezing and thawing cycle suggesting potential structural changes that facilitated intracellular staining (Supplemental Fig. 2). A similar scatter pattern was observed in cryopreserved PBMC samples.

In summary, based on cell detection rates and staining performance, only the fixed unfrozen sample exhibited results comparable to the fresh sample, while the cryopreserved sample showed the most distinct staining and scatter patterns.

### Stability of fixed unfrozen samples preserved for four weeks at 4 °C

**Fig. 4:**
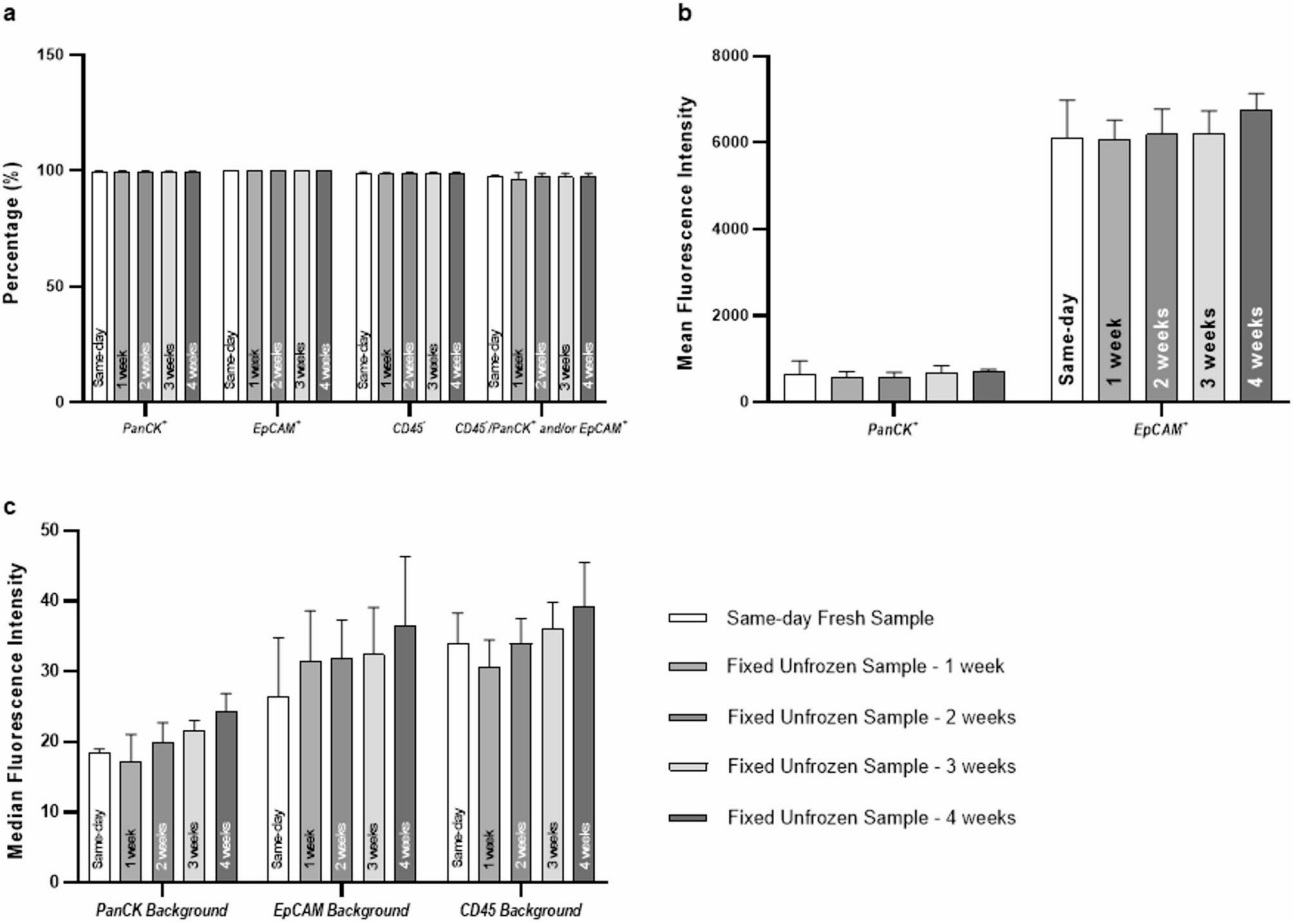
Flow cytometry analysis of PanCK, EpCAM, CD45 expression in fixed HepG2 cells stored at 4 °C for one, two, three, or four weeks, compared to same-day fresh sample. (**a**) Comparison of PanCK^**+**^, EpCAM^**+**^, CD45^**−**^ rate, and the “CTC” detection rate (CD45⁻/PanCK⁺ and/or EpCAM⁺) across five groups. (**b**) Comparison of mean fluorescence intensity of PanCK and EpCAM across five groups. (**c**) Comparison of auto fluorescence background of PanCK, EpCAM, CD45 in unstained samples across five groups. This experiment was repeated in three independent replicates. Statistical significance was determined using Dunn’s multiple comparisons tests after Kruskal-Wallis tests [*, *P* < 0.05; **, *P* < 0.01; ***, *P* < 0.001; ****, *P* < 0.0001].

As our results showed the fixed unfrozen sample was the most comparable to the fresh sample, we further evaluated its stability of fixed unfrozen samples over time. Each group of samples were stored for one, two, three, or four weeks and analyzed by flow cytometry to compare with same-day fresh sample (Supplemental Table 4). There were no significant differences in individual antibody staining (PanCK or EpCAM positivity rate, CD45 negativity rate), the combined staining-based “CTC” detection rate (CD45⁻/PanCK⁺ and/or EpCAM⁺), or the mean fluorescence intensity of PanCK and EpCAM among all five groups (Fig. 4a and b).

Besides, we observed a trend of gradual increase in auto fluorescence background over time among fixed unfrozen samples (Fig. 4c); however, this increase was not statistically significant. Storage beyond four weeks at 4 °C would not be recommended due to more increased auto fluorescence background or marker degradation, however, fixed unfrozen samples appear to maintain stable marker retention for at least four weeks.

### Fixed samples yield comparable cell recovery rates to fresh samples

**Fig. 5:**
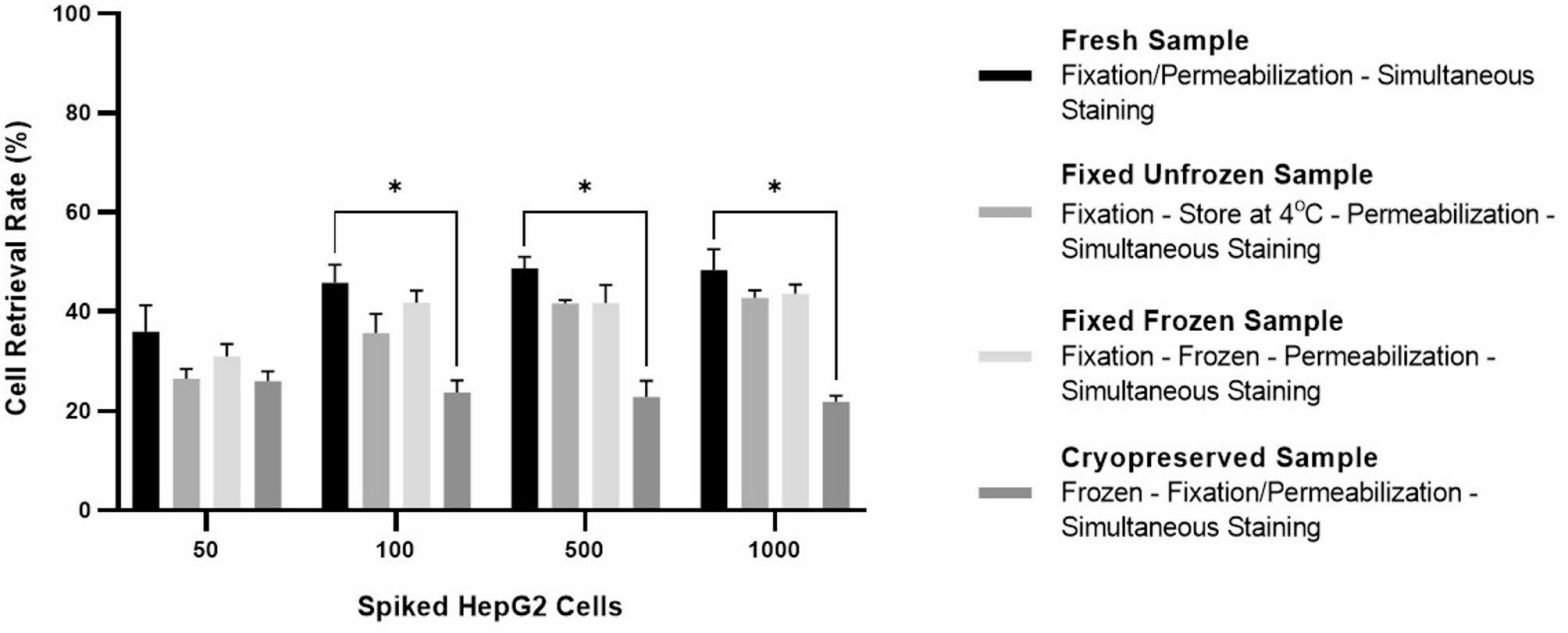
Comparison of cell retrieval rates across four sample preparation methods (fresh, fixed unfrozen, fixed frozen, and cryopreserved) using HepG2 cells spiked at four different cell numbers (50, 100, 500, and 1,000) into PBMCs. This experiment was repeated in four independent replicates. Statistical significance was determined using Dunn’s multiple comparisons tests after Kruskal-Wallis tests [*, *P* < 0.05; **, *P* < 0.01; ***, *P* < 0.001; ****, *P* < 0.0001].

To further evaluate whether fixed samples could serve as a practical alternative to fresh samples in a clinical setting, we compared cell retrieval rates across four sample preparation methods: fresh, cryopreserved, fixed frozen, and fixed unfrozen (Fig. 5 and Supplemental Table 5). To control variability introduced by enrichment, which was standardized across all groups, HepG2 cells were spiked directly into PBMCs instead of whole blood sample. Each sample preparation method was repeated four times, each time including four spiked samples containing 50, 100, 500, and 1,000 HepG2 cells, respectively. During flow cytometry analysis, single nucleated cells were first gated as CD45 negative, and among them, only those positive for either PanCK, EpCAM, or both (CD45⁻/PanCK⁺ and/or EpCAM⁺) were counted as HepG2 cells.

When only 50 cells were spiked, retrieval rates did not significantly differ among groups, ranging from 26.00% (cryopreserved sample) to 36.00% (fresh sample). However, at higher cell inputs (100–1,000), cryopreserved samples consistently showed significantly lower retrieval rates compared to fresh samples, with an average recovery of 23.67% versus 44.71%, and a maximum difference of 26.53% when 1,000 cells were spiked. In contrast, both fixed frozen and fixed unfrozen samples showed no statistically significant differences in retrieval rates compared to the fresh sample across all spiked cell numbers, although a trend toward lower recovery was observed in both fixed samples. Average cell retrieval rates were 39.51% for fixed frozen and 36.66% for fixed unfrozen samples, with the largest observed differences from fresh samples being 7% and 10%, respectively. Additionally, no significant difference was found between fixed unfrozen and fixed frozen samples across all spiked cell numbers.

These results suggest that fixed samples – whether frozen or unfrozen – maintain acceptable cell recovery and are suitable alternatives to fresh samples for CTC analysis by flow cytometry. While same-day sorting of fresh samples yields the highest recovery, fixed samples demonstrated only a modest decrease in retrieval (7% − 10%) and offer greater flexibility for downstream analysis and clinical workflow.

### Evaluation of fixed sample Preparation method for detecting CTCs in patients with hepatocellular carcinoma

To assess CTCs detection using fixed sample preparation in hepatocellular carcinoma (HCC) patient blood samples, we analyzed peripheral blood from ten patients following CD45-based depletion enrichment. After enrichment, all samples were fixed, stored, and later stained simultaneously with PanCK, EpCAM, and CD45 antibodies before flow cytometry sorting. CTCs were defined as CD45 negative and either PanCK or EpCAM positive or both (CD45⁻/PanCK⁺ and/or EpCAM⁺). A representative gating and sorting strategy is provided in Supplemental Fig. 3.

Patient tumor features and CTC detection results are summarized in Table 1, including BCLC (Barcelona Clinic Liver Cancer) stage, number of lesions, and the largest lesion size at the time of blood collection. CTCs were successfully detected in all ten blood samples including those with early-stage HCC, with an average of nine CTCs per 5 ml of blood.

**Table 1:** Tumor features and CTC detection of HCC patients.

Patient ID	Blood Collection Time	BCLC stage	Number of lesions	Largest lesion size (cm)	CTC (per 5 ml)	CTNNB1 Codon 32–37 Mutation%	
						Plasma cfDNA	CTC DNA
HCCAK503	Pre-treatment	C	10	5.6	16	NA	NA
HCCAK516	Pre-transplantation	A	1	3.3	11	NA	NA
HCCAK519	Pre-transplantation	B	3	3.4	2	NA	NA
HCCAK484	Post-TACE	B	1	2.8	2	NA	NA
HCCAK521*	Pre-TACE	B	4	4	6	**BLOD (QNS)** ^**+**^	**1.14%**,** 1.25%**
HCCAK526*	Pre-TACE	B	1	6.4	28	**BLOD (QNS)** ^**+**^	**0.48%**,** 1.12%**
HCCAK505	Post-TACE	0	1	1.6	11	NA	NA
HCCAK520	Pre-TACE	A	1	3	6	NA	NA
HCCAK493	Pre-TACE	B	1	5.3	3	NA	NA
HCCAK489	Pre-transplantation	B	1	2.4	7	NA	NA

*CTC and plasma DNA samples from these two patients were tested using ddPCR with a gene mutation panel.

^+^QNS, quantity not sufficient due to low DNA concentration, < 500 copies DNA input was used for assay. For CTNNB1 Codon T41A and S45F, both mutations were undetected due to QNS in cfDNA and CTC DNA samples.

The mutation% shown were calculated by the total mutant copies over the total CTNNB1 copies. These two results were verified in a repeat run.

To evaluate molecular integrity and confirmation of tumor origin, the whole genome DNA was extracted and amplified from sorted CTCs. In two tested samples, CTC-derived DNA revealed tumor-specific CTNNB1 mutations (codons 32–37 and 41–45), while the matched plasma cfDNA from the same patients tested negative for CTNNB1 mutations (Supplemental Table 6).

This small patient cohort demonstrates that CTCs can be efficiently detected and sorted after fixation and storage. Furthermore, the detection of tumor-specific mutations in CTC-derived DNA suggests that fixed CTCs are valuable for downstream molecular analysis compared to circulating free DNA.

## Materials and methods

### Cell culture and reagents

The hepatocellular cell line, HepG2 (ATCC, Cat#HB-8065) was cultured in Eagle’s Minimum Essential Medium (EMEM) (Quality Biological, Cat#112-018-101) with L-glutamine supplemented with 10% heat-inactivated fetal bovine serum (FBS) (Gibco, Cat#A56708-01) and 1% penicillin/streptomycin (Gibco, Cat#15140122) at 37 °C with 5% CO2. Cell line is tested mycoplasma-free using the MycoAlert Plus Mycoplasma Detection Kit (Lonza, Cat# LT07-707).

### Flow cytometry analysis

#### Live cell staining

HepG2 cells were harvested after trypsinization, washed, and divided into five tubes, then suspended in 2% FBS/PBS per tube for staining. One tube was left unstained as a negative control, while the remaining tubes were stained with Pan Cytokeratin antibody (Thermofisher, Cat#MA5-28574, 1:25), APC anti-EpCAM antibody (Abcam, Cat#ab239288, 1:100), brilliant violet 785 anti-human CD45 antibody (BioLegend, Cat#304048, 1:50), or a combination of these three antibodies at the same concentrations. After 30 min of incubation, all samples were washed twice with 2% FBS/PBS, then re-suspended in 2% FBS/PBS with DCV and incubated for an additional 15 min at 37 °C. Just before flow cytometry analysis, all samples were stained with 1 µl/ml of propidium iodide (Invitrogen, Cat#3566).

#### Serial staining with fixation and permeabilization

As described above for live cell staining, harvested HepG2 cells were divided into five tubes, re-suspended in 2% FBS/PBS. Three tubes were stained with either EpCAM antibody (1:100), CD45 antibody (1:50), or a combination of these two antibodies at the same concentrations, while the remaining two tubes remained unstained. After 30 min of incubation, all samples were washed twice with 2% FBS/PBS, then fixed with freshly prepared 4% paraformaldehyde (PFA) (Electron Microscopy Sciences, Cat#15714-S) for 10 min at room temperature.

Following fixation, all samples were washed twice and re-suspended in 1X Perm/Wash buffer (BD, Cat#554723). One of the two unstained tubes was left unstained as a negative control, while the other was stained with PanCK antibody (1:100). In addition, the sample previously stained with both EpCAM and CD45 antibodies was further stained with PanCK antibody (1:100). After another 30 minutes’ incubation, all samples were washed twice with 1X Perm/wash buffer. Finally, all samples were re-suspended in 2% FBS/PBS with 2 µg/ml of DAPI, and kept on ice until flow cytometry analysis.

#### Simultaneous staining with fixation and permeabilization

If HepG2 cells were cryopreserved in freezing media (FBS: DMSO = 9:1), they were thawed, washed, and subsequently fixed with 4% PFA. If HepG2 cells were freshly harvested, they were washed once then fixed with 4% PFA. Following fixation, all samples were washed twice, divided into five tubes, and re-suspended in 100ul of 1X Perm/wash buffer per tube. One tube was left unstained as a negative control, while the remaining tubes were stained with PanCK antibody (1:100), EpCAM antibody (1:100), CD45 antibody (1:50), or a combination of all three antibodies at the same concentrations. After incubation, all samples were washed twice with 1X Perm/wash buffer, then re-suspended in 2% FBS/PBS with 2 µg/ml of DAPI, and kept on ice until flow cytometry analysis.

#### Fixed samples with simultaneous staining

HepG2 cells were harvested and washed once prior to fixation. After fixation with 4% PFA, cells were washed twice, and either re-suspended in freezing media (FBS: DMSO = 9:1) and stored at -80 °C (fixed frozen sample), or re-suspended in eBioscience flow cytometry staining buffer (Invitrogen, Cat#00-422-57) and stored at 4 °C (fixed unfrozen sample).

Following thawing and one wash, the fixed frozen sample was divided into five tubes, and each re-suspended in 1X Perm/wash buffer and subjected to the simultaneous staining protocol as previously described. Similarly, the fixed unfrozen sample was divided into five tubes, centrifuged, and re-suspended in 1X Perm/wash buffer per tube before continuing with the same combined staining procedure.

#### Cell spiking

Freshly harvested HepG2 cells were stained with 1 µg/mL of propidium iodide for live HepG2 cell sorting. 50, 100, 500, or 1,000 HepG2 cells were respectively sorted using fluorescence-activated cell sorting (FACS) and spiked into 100,000 PBMCs in 500 µl of 2% FBS/PBS. Spiked samples underwent different sample preparation methods as described above (fresh, cryopreserved, fixed frozen, fixed unfrozen) and were then simultaneously stained with PanCK, EpCAM, CD45 antibodies using the previously described protocols prior to flow cytometry analysis.

All samples were analyzed by MoFlo-XDP (Beckman Coulter, 4 lasers, 17 colors) within 1 h of staining. Data collection and analysis were conducted using Summit version 4.3.01 (Beckman Coulter, https://www.beckman.com/) and FCS Express version 7 (De Novo Software, Pasadena, CA; https://denovosoftware.com/).

### Blood sample collection and processing

This study was approved by the Johns Hopkins Medicine Institutional Review Board (Baltimore, Maryland, United States) and was conducted in accordance with the ethics principles of the Declaration of Helsinki. Informed consent was obtained from all patients and healthy donors prior to enrollment. All blood samples were collected in a 10-ml Vacutainer EDTA tube (BD, Cat# 366643) and processed within 2 h after collection.

#### Circulating tumor cells (CTCs) collection from hepatocellular carcinoma (HCC) patients

Peripheral blood samples (10 ml) from HCC patients (*n* = 10) were collected for CTC analysis. Briefly, 500 µl of RosetteSep Human CD45 depletion cocktail (Stemcell Technologies, Cat#15162) was added to each 10 ml blood sample for CTC negative enrichment following the manufacture’s instruction. The blood sample was then diluted with 2% FBS/PBS, centrifuged with a density gradient medium, and washed to enrich CTCs. The sample was then incubated with ammonium chloride solution (Stemcell Technologies, Cat#07800) for red blood cell lysis. After RBC lysis, the enriched CTCs were fixed with freshly prepared 4% PFA, washed twice. Enriched CTCs were re-suspended in 1 ml of freezing media (FBS: DMSO = 9:1) for storage at -80 °C or 1 ml of eBioscience flow cytometry staining buffer (Invitrogen, Cat# 00-4222-57) for storage at 4 °C for future analysis.

#### Plasma isolation from hepatocellular carcinoma (HCC) patients

An additional 10 ml peripheral blood sample was collected from each HCC patient (*n* = 10). Plasma was centrifuged at 2000 x g for 10 min. The collected plasma was then subjected to a second centrifugation at 10,000 x g for 10 min to remove any remaining cells and debris. The final plasma sample was aliquoted into cryogenic tubes and stored at -80 °C for future use.

#### Peripheral blood mononuclear cells (PBMCs) isolation from healthy donors

Peripheral blood samples (10 ml) from healthy donors (*n* = 5) were collected for PBMC isolation. The blood sample was then diluted with PBS, centrifuged with a density gradient medium, and washed twice to harvest PBMCs. The isolated PBMCs were then re-suspended in freezing media (FBS: DMSO = 9:1) and stored at -80 °C for future use.

### Fluorescence-activated cell sorting (FACS)

Fixed CTCs were centrifuged, re-suspended in 1X Perm/Wash buffer and labeled with PanCK antibody (1:100), EpCAM antibody (1:100) and CD45 antibody (1:50). After washed twice with 2 ml of 1X Perm/Wash buffer, CTCs were re-suspended in 500 µl of 1X PBS with 2 µg/ml of DAPI, and kept on ice until FACS.

During FACS analysis, single cells were first identified by gating on side scatter (SSC) and forward scatter (FSC) to exclude doublets and debris. The criteria for CTCs identification were as follows: cells were negative for the leukocyte marker CD45 and positive for at least one epithelial marker – PanCK and/or EpCAM (CD45⁻/PanCK⁺ and/or EpCAM⁺) based on CD45/EpCAM/PanCK gating. HepG2 cells single-stained for PanCK or EpCAM were used to define the positive gates for epithelial markers, while PBMCs single-stained for CD45 established the negative gate for CD45. Unstained HepG2 cells and PBMCs were included to assess background fluorescence, and combined staining was performed to control potential marker co-expression. CTCs isolated by these criteria were subsequently sorted using the MoFlo-XDP (Beckman Coulter, 4 lasers, 17 colors), collected in 1X PBS, snap-frozen, and stored at -80 °C for future analysis.

### Droplet digital PCR

#### CTNNB1 codon 32–37 hotspot mutations by droplet digital PCR (ddPCR)

Mutations in codons 32–37 (hg38 Chr3:41,224,606 − 41,224,623) of exon 3 of the CTNNB1 gene were detected by the CTNNB1 32–37 droplet digital PCR kit (JBS Science, Inc., Doylestown, PA) according to the manufacturer’s protocol. The PCR utilizes a drop-off approach to identify mutations located between codons 32–35 and codons 36 − 7. Droplet generation was performed using the Naica Crystal PCR system (Stilla Technologies). The reaction mixture contained 5 µl PerfeCTa MultiPlex qPCR ToughMix (QuantaBio), 2.5 µl of 25x assay mix, DNA template (maximum of 5,000 copies) adjusted for a final volume of 25 µl. The cycling protocol was as follows 1 cycle at 95 °C 5 min, followed by 50 cycles of 95 °C 10s, 80 °C 5s, 60 °C 1s. The ddPCR data was analyzed using the CrystalMiner for the Naica System version 4.0 software (Stilla Technologies, Villejuif, France; https://www.stillatechnologies.com/), and positive and negative controls were used to determine the thresholds for each run.

#### CTNNB1 T41A/S45F mutations by DdPCR

The CTNNB1 Duplex T41A and S45F ddPCR kit (JBS) was used to assess the mutation at codon 41 (hg38, Chr3:41224633 A > G) and codon 45 (hg38, Chr3: 41224646 C > T according to the manufacturer’s protocol. Droplet generation was performed using the Naica Crystal PCR system (Stilla Technologies). The reaction mixture contained 5 µl PerfeCTa MultiPlex qPCR ToughMix (QuantaBio), 2.5 µl of 25x assay mix, DNA template (maximum of 5,000 copies) adjusted for a final volume of 25 µl. The cycling protocol was as follows 1 cycle at 95 °C 5 min, followed by 50 cycles of 95 °C 10s, 56 °C 10s, 72 °C 10s. The ddPCR data was analyzed using the CrystalMiner for the Naica System version 4.0 software (Stilla Technologies, Villejuif, France; https://www.stillatechnologies.com/), and positive and negative controls were used to determine the thresholds for each run.

### Statistical analysis methods

Statistical differences between two groups were analyzed using Wilcoxon rank sum tests. For comparisons among multiple groups, Kruskal-Wallis tests were performed, followed by Dunn’s multiple comparisons tests to compare each group to the fresh sample. Results are presented as medians with corresponding min-max ranges, and a P value less than 0.05 was considered statistically significant. Statistical analyses were performed using GraphPad Prism version 10.6.0 for Windows (GraphPad Software, Boston, MA, USA; https://www.graphpad.com/).

## Discussion

CTCs have emerged as a significant biomarker for cancer detection and disease monitoring^3,4,8,24^. However, their clinical utility is limited by logistical challenges, including the need for time-sensitive sample processing and immediate downstream analysis. In this study, we addressed these limitations by establishing a fixation-compatible workflow for CTC detection and sorting using flow cytometry, optimized for HCC and broadly applicable to other solid tumors.

A few key findings from our study should be highlighted. First, simultaneous staining of cell surface and intracellular markers after fixation yields staining performance comparable to, or better than, the traditional serial staining method. Specifically, this method modestly improved EpCAM signal intensity in HepG2 cells, while reducing wash steps and potential cell loss during processing. Additionally, early fixation may preserve fragile cells that are prone to apoptosis after blood sample collection and allows greater flexibility in centrifugation condition, further reducing the cell loss compared to live-cell handling.

Second, we show that fixed samples, particularly fixed unfrozen samples, perform comparably to fresh samples in terms of CTCs detection. Although a slight reduction in recovery (7–10%) was observed compared to same-day fresh sample processing, this trade-off is offset by substantial logistical advantages. Notably, storage conditions (fixed frozen vs. fixed unfrozen) did not significantly affect retrieval efficiency, indicating stability of fixed samples even after freeze-thaw cycles. In contrast, our results show that unfixed cryopreserved samples deviate most from fresh samples in staining performance and cell retrieval, making them suboptimal for CTC analysis. Although cryopreservation is used in other studies, our data indicates that fixation should occur before freezing to ensure reliable downstream analysis. Post-thaw fixation increases cell membrane fragility – as reflected in altered scatter patterns and staining differences – and leads to greater cell loss and more false-positive CD45 staining on epithelial cells. These findings suggest that fixation prior to freezing is preferable to conventional cryopreservation for preserving CTC integrity.

We also validate the protocol in clinical samples. CTCs were successfully detected and sorted from all ten HCC patient samples, including those with early-stage disease. Importantly, CTC-derived DNA from two patient samples revealed tumor-specific gene mutation (CTNNB1) there were not detected in matched plasma cfDNA, suggesting that potential of fixed CTCs to provide complementary or superior genomic information than cell-free DNA alone.

The proposed fixed sample for flow cytometry addresses a critical barrier in CTC research: the need for standardized, scalable, and logistically feasible workflow. The ability to store fixed samples for up to four weeks at 4 °C without compromising recovery, enables batching and flexibility. However, a gradual increase in auto fluorescence was observed over time, suggesting that if the timing of downstream analysis is uncertain, fixed frozen samples may be the more optimal storage choice.

Despite the positive findings, our study has certain limitations. The experiment validation was limited to a single cell line (HepG2) and PMBCs and clinical feasibility was shown in a small number of patients. Additional studies incorporating more diverse cell lines and larger patient samples will be important for validating the protocol’s generalizability. Moreover, our evaluation was based on a single specific enrichment method (RosetteSep Human CD45 Depletion) and the impact of upstream steps including density gradient separation and RBC lysis was not assessed. Ultimately, developing a more reliable, specific, and efficient CTC detection method with minimal cell loss during enrichment and staining remains a critical goal for future CTC research.

In conclusion, our study presents a robust, practical CTC sample preparation and detection method that enables simultaneous analysis of surface and intracellular markers in fixed cells. It supports reliable CTC detection, efficient sample handling, and high-quality molecular readouts, offering a viable alternative to fresh sample workflows for biomarker studies and translational research in HCC.

## Supplementary Information

Below is the link to the electronic supplementary material.

Supplementary Material 1

## Data Availability

The datasets used and analyzed during the current study are available from the corresponding author on reasonable request.
