# Supplementary material for: A fixation-compatible protocol for intracellular and surface marker-based detection of circulating tumor cells in hepatocellular carcinoma
Source: Sci Rep. 2025 Nov 7;15:39116. doi: 10.1038/s41598-025-23698-1 (PMC12594790; doi:10.1038/s41598-025-23698-1)
Supplement: Supplementary file 1 — Supplementary Material 1 [file 41598_2025_23698_MOESM1_ESM.docx]

**Supplemental Data**

**Supplemental Figure 1**. Workflow of four sample preparation methods. (a) Fresh sample: Cells are harvested, fixed, permeabilized, and simultaneously stained with cell surface and intracellular antibodies. (b) Cryopreserved sample: Cells are harvested, frozen, and stored at -80°C. On the day of analysis, cells are thawed, fixed, permeabilized, and simultaneously stained with cell surface and intracellular antibodies. (c) Fixed frozen sample: Cells are harvested, fixed, frozen, and stored at -80°C. After thawing, cells are permeabilized and simultaneously stained with cell surface and intracellular antibodies. (d) Fixed unfrozen sample: Cells are harvested, fixed, and stored at 4°C. On the day of analysis, cells are permeabilized and simultaneously stained with cell surface and intracellular antibodies.


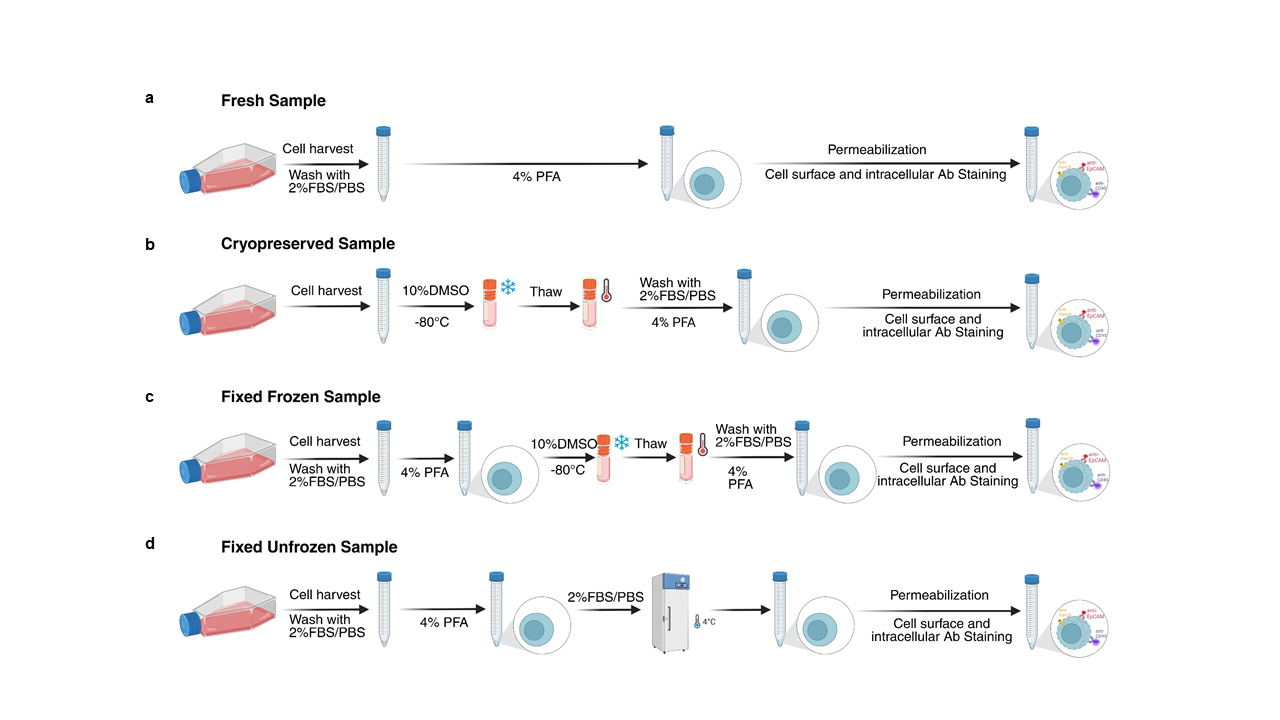


**
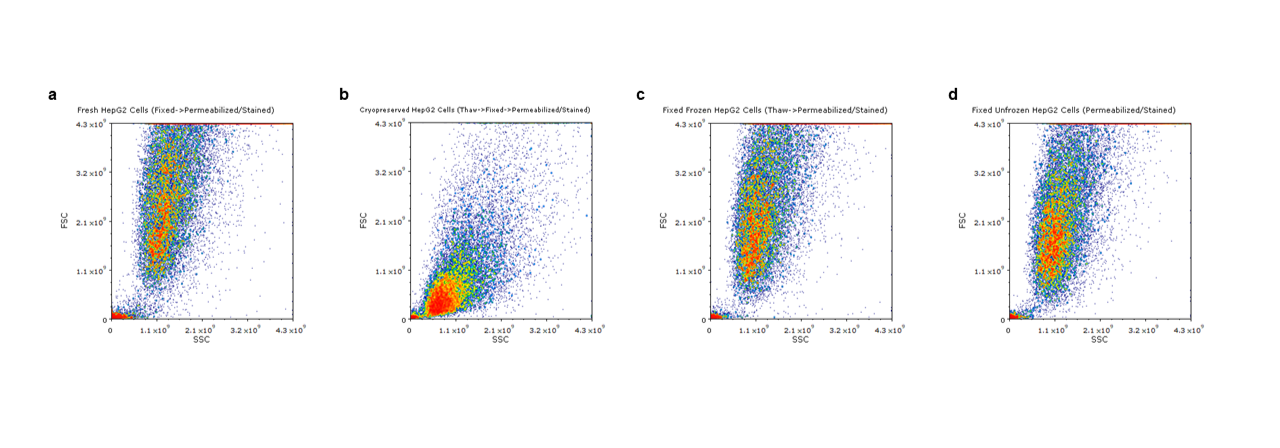
**

**Supplemental Figure 2**. Scatter pattern comparison across four sample preparation methods. (a) Fresh HepG2 cells. (b) Cryopreserved HepG2 cells. (c) Fixed frozen HepG2 cells. (d) Fixed unfrozen HepG2 cells.


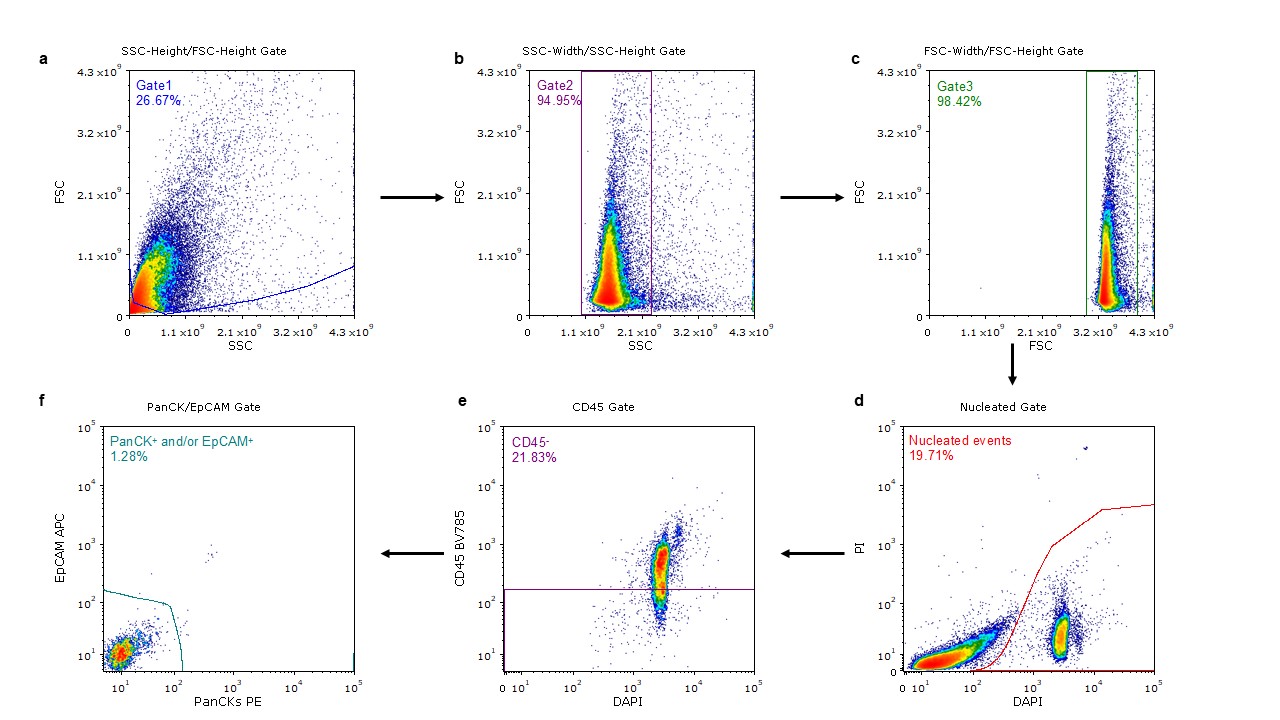


**Supplemental Figure 3**. Representative sorting strategy on fixed CTC samples from HCC patients. (a - c) Side scatter (SSC) and forward scatter (FSC) gating to identify single cells and exclude cell debris, doublets, or aggregates. (d) Nucleated cells are selected using DAPI gating from FSC/SCC-selected events. (e) CD45 negative nucleated cells are identified using CD45 gating (CD45 threshold established from PMBCs CD45 staining). (f) CTCs are defined as CD45 negative and either PanCK or EpCAM positive or both (CD45⁻/PanCK⁺ and/or EpCAM⁺). EpCAM and PanCK thresholds are determined by HepG2 cells PanCK and EpCAM staining.

| Parameters | groups | Median | Min, Max | P value |
| --- | --- | --- | --- | --- |
| PanCK^+^ (%) | Serial Staining | 95.48 | 79.98, 96.42 | 0.2000 |
|  | Simultaneous Staining | 98.09 | 95.00, 99.04 |  |
| EpCAM^+^ (%) | Serial Staining | 99.62 | 99.44, 99.82 | 0.1143 |
|  | Simultaneous Staining | 99.83 | 99.81, 99.87 |  |
| CD45^-^ (%) | Serial Staining | 99.86 | 99.75, 99.96 | 0.0286 |
|  | Simultaneous Staining | 98.96 | 98.79, 99.12 |  |
| CD45⁻/PanCK⁺  and/or EpCAM⁺ (%) | Serial Staining | 98.93 | 98.50, 99.36 | >0.9999 |
|  | Simultaneous Staining | 98.58 | 98.57, 98.59 |  |
| PanCK^+^ MFI | Serial Staining | 532.10 | 313.10, 746.72 | 0.6857 |
|  | Simultaneous Staining | 510.60 | 358.46, 785.74 |  |
| EpCAM^+^ MFI | Serial Staining | 6264.00 | 6154.51, 6431.01 | 0.0286 |
|  | Simultaneous Staining | 7234.00 | 7097.50, 7545.07 |  |
| PanCK^+^ SI | Serial Staining | 16.34 | 9.30, 28.21 | 0.8571 |
|  | Simultaneous Staining | 18.98 | 15.18, 27.50 |  |
| EpCAM^+^ SI | Serial Staining | 226.70 | 187.97, 256.77 | 0.2857 |
|  | Simultaneous Staining | 262.90 | 219.63, 296.03 |  |

**Supplemental Table 1.** Comparison of Staining Effect between Serial Staining and Simultaneous Staining using HelpG2 cells.

|  | **Sample Group – HepG2 Cells** | | | |
| --- | --- | --- | --- | --- |
| **Parameters** | **Fresh** | **Cryopreserved** | **Fixed Frozen** | **Fixed Unfrozen** |
| **PanCK⁺ (%)** |  |  |  |  |
| Median (Min, Max) | 98.09 (95.00, 99.04) | 98.03 (96.78, 99.13) | 98.58 (97.04, 99.42) | 99.04 (98.68, 99.44) |
| P value | *REF* | 0.6857 | 0.4857 | 0.3429 |
| **EpCAM⁺ (%)** |  |  |  |  |
| Median (Min, Max) | 99.83 (99.80, 99.87) | 99.91 (99.50, 99.84) | 99.91 (99.89, 99.94) | 99.94 (99.86, 99.97) |
| P value | *REF* | 0.0857 | 0.0286 | 0.0571 |
| **CD45⁻ (%)** |  |  |  |  |
| Median (Min, Max) | 98.96 (98.79, 99.12) | 92.12 (88.86, 95.80) | 89.13 (86.27, 94.03) | 99.15 (98.42, 99.51) |
| P value | *REF* | 0.0286 | 0.0347 | 0.8857 |
| **CD45⁻/PanCK⁺**  **and/or EpCAM⁺ (%)** |  |  |  |  |
| Median (Min, Max) | 98.58 (98.57, 98.59) | 86.34 (82.66, 90.02) | 87.10 (84.51, 89.69) | 98.27 (97.67, 98.87) |
| P value | *REF* | 0.3333 | 0.3333 | >0.9999 |
| **PanCK⁺ MFI** |  |  |  |  |
| Median (Min, Max) | 510.60 (358.46, 785.74) | 1339.00 (1302.98, 1485.25) | 309.90 (265.54, 428.06) | 354.00 (327.77, 410.51) |
| P value | *REF* | 0.0286 | 0.1143 | 0.3429 |
| **EpCAM⁺ MFI** |  |  |  |  |
| Median (Min, Max) | 7234.00 (7097.50, 7545.07) | 5136.00 (4435.18, 5803.73) | 5975.00 (5228.69, 6845.67) | 6009.00 (5319.91, 6914.53) |
| P value | *REF* | 0.0089 | 0.0938 | 0.1349 |
| **PanCK⁺ SI** |  |  |  |  |
| Median (Min, Max) | 18.98 (15.18, 27.50) | 58.65 (55.09, 68.40) | 12.74 (10.34, 17.82) | 14.95 (9.79, 24.17) |
| P value | *REF* | 0.0286 | 0.1143 | 0.4571 |
| **EpCAM⁺ SI** |  |  |  |  |
| Median (Min, Max) | 262.90 (219.63, 296.03) | 165.30 (157.54, 165.71) | 140.10 (78.27, 211.33) | 182.40 (109.48, 262.96) |
| P value | *REF* | 0.0286 | 0.0286 | 0.3143 |

**Supplemental Table 2.** Comparison of Staining Effect Across Four Sample Preparation Methods (fresh, cryopreserved, fixed frozen, and fixed unfrozen) using HepG2 cells.

|  | **Sample Group - PBMCs** | | | |
| --- | --- | --- | --- | --- |
| **Parameters** | **Fresh** | **Cryopreserved** | **Fixed Frozen** | **Fixed Unfrozen** |
| **CD45⁺ (%)** |  |  |  |  |
| Median (Min, Max) | 99.68 (99.64, 99.71) | 99.6 (99.53, 99.66) | 99.34 (99.31, 99.36) | 99.33 (99.31, 99.35) |
| P value | *REF* | >0.9999 | 0.2427 | 0.1532 |
| **CD45⁺ MFI** |  |  |  |  |
| Median (Min, Max) | 860.60 (810.59, 940.58) | 911.70 (858.39, 965.04) | 895.90 (875.39, 916.49) | 877.00 (847.19, 906.79) |
| P value | *REF* | >0.9999 | >0.9999 | >0.9999 |
| **CD45⁺ SI** |  |  |  |  |
| Median (Min, Max) | 38.24 (36.48, 40.00) | 33.35 (31.83, 34.88) | 39.05 (38.15, 39.95) | 35.07 (34.26, 35.88) |
| P value | *REF* | 0.3972 | >0.9999 | 0.9183 |

**Supplemental Table 3.** Comparison of Staining Effect Across Four Sample Preparation Methods (fresh, cryopreserved, fixed frozen, and fixed unfrozen) using PBMCs.

|  | **Sample Group – HepG2 Cells** | | | | |
| --- | --- | --- | --- | --- | --- |
| **Parameters** | **Fresh** | **Fixed Unfrozen 1 wk** | **Fixed Unfrozen 2 wks** | **Fixed Unfrozen 3 wks** | **Fixed Unfrozen 4 wks** |
| **PanCK⁺ (%)** |  |  |  |  |  |
| Median (Min, Max) | 99.60 (99.19, 99.90) | 99.71 (99.30, 99.88) | 99.72 (99.40, 99.86) | 99.71 (99.48, 99.87) | 99.74 (99.62, 99.86) |
| P value | *REF* | 0.7729 | 0.6962 | 0.7811 | 0.6177 |
| **EpCAM⁺ (%)** |  |  |  |  |  |
| Median (Min, Max) | 99.97 (99.96, 100.00) | 99.97 (99.94, 99.98) | 99.97 (99.95, 100.00) | 99.95 (99.87, 99.99) | 99.95 (99.89, 99.98) |
| P value | *REF* | 0.6492 | 0.9046 | 0.4018 | 0.3381 |
| **CD45⁻ (%)** |  |  |  |  |  |
| Median (Min, Max) | 99.04 (98.65, 99.63) | 98.57 (97.64, 99.11) | 98.93 (98.38, 99.30) | 98.91 (98.51, 99.67) | 99.00 (98.30, 99.53) |
| P value | *REF* | 0.3893 | 0.9907 | 0.9831 | 0.9998 |
| **CD45⁻/PanCK⁺**  **and/or EpCAM⁺ (%)** |  |  |  |  |  |
| Median (Min, Max) | 97.39 (96.89, 97.89) | 97.94 (93.18, 97.17) | 97.83 (95.73, 98.55) | 97.30 (95.54, 98.77) | 97.93 (95.36, 98.53) |
| P value | *REF* | 0.8000 | >0.9999 | >0.9999 | 0.8000 |
| **PanCK⁺ MFI** |  |  |  |  |  |
| Median (Min, Max) | 637.30 (347.39, 923.73) | 582.70 (389.36, 741.06) | 607.00 (418.73, 704.50) | 701.70 (418.09, 870.42) | 704.90 (638.54, 768.34) |
| P value | *REF* | >0.9999 | >0.9999 | >0.9999 | >0.9999 |
| **EpCAM⁺ MFI** |  |  |  |  |  |
| Median (Min, Max) | 6123.00 (5333.35, 6864.82) | 5981.00 (5679.56, 6950.36) | 5910.00 (5679.56, 6950.36) | 5902.00 (5750.66, 6899.77) | 6822.00 (6262.44, 7274.46) |
| P value | *REF* | >0.9999 | 0.4762 | 0.4762 | 0.4762 |
| **Auto Fluorescence**  **Background of PanCK** |  |  |  |  |  |
| Median (Min, Max) | 18.40 (17.98, 18.82) | 17.19 (13.11, 21.54) | 17.98 (17.98, 23.58) | 22.54 (19.68, 22.54) | 24.67 (21.54, 27.00) |
| P value | *REF* | >0.9999 | >0.9999 | 0.8637 | 0.1636 |
| **Auto Fluorescence**  **Background of EpCAM** |  |  |  |  |  |
| Median (Min, Max) | 26.47 (20.59, 32.34) | 28.25 (25.81, 40.54) | 29.55 (27.00, 38.75) | 30.92 (25.81, 40.54) | 33.84 (27.00, 48.56) |
| P value | *REF* | >0.9999 | >0.9999 | >0.9999 | 0.7175 |
| **Auto Fluorescence**  **Background of CD45** |  |  |  |  |  |
| Median (Min, Max) | 33.98 (30.92, 37.04) | 32.34 (25.81, 33.84) | 35.40 (29.55, 37.04) | 35.40 (32.34, 40.54) | 38.75 (32.34, 46.42) |
| P value | *REF* | 0.6071 | 0.7500 | 0.6071 | 0.3214 |

**Supplemental Table 4.** Comparison of Staining Effect and Auto Fluorescence Background Across Five Groups (fresh sample and fixed unfrozen sample 1 week, 2 weeks, 3 weeks and 4 weeks) using HepG2 cells.

|  | **Sample Group** | | | |
| --- | --- | --- | --- | --- |
| **Parameters** | **Fresh** | **Fixed Unfrozen** | **Fixed Frozen** | **Cryopreserved** |
| **50 HepG2 cells spiked Cell Retrieval Rate (%)** |  |  |  |  |
| Median (Min, Max) | 36.00 (22.00, 46.00) | 26.50 (22.00, 32.00) | 31.00 (26.00, 38.00) | 26.00 (24.00, 32.00) |
| P value | *REF* | 0.4981 | >0.9999 | 0.2986 |
| **100 HepG2 cells spiked Cell Retrieval Rate (%)** |  |  |  |  |
| Median (Min, Max) | 47.75 (38.00, 53.00) | 35.75 (27.00, 43.00) | 41.75 (36.00, 48.00) | 23.75 (19.00, 28.00) |
| P value | *REF* | >0.9999 | >0.9999 | 0.0349 |
| **500 HepG2 cells spiked Cell Retrieval Rate (%)** |  |  |  |  |
| Median (Min, Max) | 48.70 (42.40, 53.40) | 41.65 (40.80, 43.60) | 41.70 (31.20, 47.80) | 22.87 (19.40, 29.40) |
| P value | *REF* | 0.4485 | >0.9999 | 0.011 |
| **1000 HepG2 cells spiked Cell Retrieval Rate (%)** |  |  |  |  |
| Median (Min, Max) | 48.38 (40.80, 59.30) | 42.75 (38.10, 44.80) | 43.60 (38.90, 48.00) | 21.85 (20.00, 25.40) |
| P value | *REF* | 0.4145 | 0.5464 | <0.0001 |

**Supplemental Table 5.** Comparison of Cell Retrieval Rates (%) Across Four Sample Preparation Method.

| **Subject ID** | **Sample type** | **DNA concentration**  **TapeStation (ng/ul)** | **Total CTNNB1 concentration (copy/ul)** | **CTNNB1** | | |
| --- | --- | --- | --- | --- | --- | --- |
|  |  |  |  | **Condon 32-37 mutation%** | **Condon T41A mutation %** | **Condon S45F mutation %** |
| AK521 | Serum | 0.18 | 88.13 | BLOD | BLOD | BLOD |
| AK521 | CTC | 89.2 | 303.75 | 1.14%; 1.25% | BLOD | BLOD |
| AK526 | Serum | 1.19 | 458.75 | BLOD | BLOD | BLOD |
| AK526 | CTC | 67 | 425 | 0.48%; 1.12% | BLOD | BLOD |

**Supplemental Table 6.** Plasma and CTC DNA concentration and gene mutation detection result. Two biomarker assays were performed, CTNNB1 32-37 droplet digital PCR (ddPCR), CTNNB1 T41A/S45F duplex ddPCR. All assays were performed with at least 1,500 DNA copy input, up to 5000 copies maximum for ddPCR assays. The mutation % shown above are the calculated by the total mutant copies over the total CTNNB1 copies. Samples with positive results were verified in a repeat run as shown above.

BLOD, below limit of detection.
